# Partial-arm translocations in evolution of malaria mosquitoes revealed by high-coverage physical mapping of the *Anopheles atroparvus* genome

**DOI:** 10.1186/s12864-018-4663-4

**Published:** 2018-04-23

**Authors:** Gleb N. Artemov, Semen M. Bondarenko, Anastasia N. Naumenko, Vladimir N. Stegniy, Maria V. Sharakhova, Igor V. Sharakhov

**Affiliations:** 1 Laboratory of Ecology, Genetics and Environmental Protection, Tomsk State University, 36 Lenin Avenue, Tomsk, 634050 Russia; 2 Department of Entomology, Fralin Life Science Institute, Virginia Polytechnic Institute and State University, 360 West Campus Drive, Blacksburg, VA 24061 USA

**Keywords:** Mosquito genome, Chromosome evolution, Partial-arm translocation, Fluorescence in situ hybridization, Physical mapping, Polytene chromosomes, *Anopheles atroparvus*

## Abstract

**Background:**

Malaria mosquitoes have had a remarkable stability in the number of chromosomes in their karyotype (2n = 6) during 100 million years of evolution. Moreover, autosomal arms were assumed to maintain their integrity even if their associations with each other changed via whole-arm translocations. Here we use high-coverage comparative physical genome mapping of three *Anopheles* species to test the extent of evolutionary conservation of chromosomal arms in malaria mosquitoes.

**Results:**

In this study, we developed a physical genome map for *Anopheles atroparvus,* one of the dominant malaria vectors in Europe. Using fluorescence in situ hybridization (FISH) of DNA probes with the ovarian nurse cell polytene chromosomes and synteny comparison, we anchored 56 genomic scaffolds to the *An. atroparvus* chromosomes. The obtained physical map represents 89.6% of the *An. atroparvus* genome. This genome has the second highest mapping coverage among Anophelinae assemblies after *An. albimanus*, which has 98.2% of the genome assigned to its chromosomes. A comparison of the *An. atroparvus*, *An. albimanus*, and *An. gambiae* genomes identified partial-arm translocations between the autosomal arms that break down the integrity of chromosome elements in evolution affecting the structure of the genetic material in the pericentromeric regions. Unlike *An. atroparvus* and *An. albimanus*, all chromosome elements of *An. gambiae* are fully syntenic with chromosome elements of the putative ancestral *Anopheles* karyotype. We also detected nonrandom distribution of large conserved synteny blocks and confirmed a higher rate of inversion fixation in the X chromosome compared with autosomes.

**Conclusions:**

Our study demonstrates the power of physical mapping for understanding the genome evolution in malaria mosquitoes. The results indicate that syntenic relationships among chromosome elements of *Anopheles* species have not been fully preserved because of multiple partial-arm translocations.

**Electronic supplementary material:**

The online version of this article (10.1186/s12864-018-4663-4) contains supplementary material, which is available to authorized users.

## Background

Chromosome rearrangements play a role in species’ adaptation and evolution by generating structural genomic variations [1], affecting recombination [2], and changing the pattern of gene expression [3, 4]. Although chromosome rearrangements affect all groups of living organisms, genomes of different lineages have been preferentially shaped by particular types of rearrangements. For example, evolution of angiosperm plants has been accompanied by multiplication of entire chromosome compliment (polyploidization) and subsequent diploidization of their genomes [5]. Chromosome translocations, which transfer a whole or part of a chromosome to another chromosome, have been the predominant mechanisms of karyotype evolution in vertebrates [6]. Paracentric inversions, which flip a chromosomal segment 180^o^, have been particularly frequent in many dipteran insects, including *Drosophila* and *Anopheles* species [7]. However, even different Diptera families display distinct patterns of chromosomal evolution. Karyotypes of flies evolve by centric fissions or fusions, by which chromosome arms are combined or separated from each other affecting the total number of chromosomes [8]. In contrast, karyotypes of *Anopheles* species have no variations in the number of chromosomes that is always 2n = 6 [9]. The mechanisms of the observed lineage-specificity in the patterns of genome rearrangements are not well understood.

Another unsolved problem of karyotypic evolution is the forces that preserve or destroy the integrity of individual chromosome elements over time. H. J. Muller proposed that the chromosomes of *Drosophila* species are represented by a set of six homologous chromosome arms [10] named Muller elements A–F. This nomenclature is used to identify homologous linkage groups among species within the *Drosophila* genus [8]. Similarly, the term “chromosome elements” has been used to define five chromosomal arms that are homologous across *Anopheles* species. The chromosomal arms in *An. gambiae* are named as follows: X = element 1 (e1), 2R = e2, 2L = e3, 3R = e4, and 3L = e5 [11, 12]. Chromosome elements of *D. melanogaster* and *An. gambiae* have only limited homology indicating that multiple inter-arm rearrangements have been accumulated since the split of the two genera about 250 million years ago (MYA) [13]. The most conserved pair of chromosomal arms, 2L of *D. melanogaster* (Muller element B) and 3R of *An. gambiae* (chromosome element 4), share 76% of the orthologs and 95% of microsynteny blocks. Physical genome mapping demonstrated that even within genus *Drosophila* a perfect one-to-one correspondence between the Muller elements and chromosome arms has been occasionally violated by pericentric inversions [8].

A pattern of chromosome rearrangements between *An. gambiae* and other mosquito species, including *An. albimanus, An. atroparvus, An. funestus, An. sinensis,* and *An. stephensi,* has been analyzed using cytogenetic physical maps and partially mapped genome assemblies [11, 12, 14–24]. These studies demonstrated that autosomal arms in *Anopheles* exchange between chromosomes via whole-arm translocation. In addition, numerous paracentric inversions reshuffle the gene order within chromosome elements. At that time, it was concluded that unlike *Drosophila*, *Anopheles* chromosomal arms evolve as intact elements [24]. However, a more recent comparative analysis of nearly complete genome maps of *An. albimanus* and *An. gambiae* detected genetic exchanges between chromosome elements 2 and 4 and between chromosome elements 3 and 5 [25]. These data suggest that inter-arm rearrangements occurred since the two subgenera, Nyssorhynchus (*An. albimanus*) and Cellia (*An. gambiae*), diverged about 100 MYA. To obtain more detailed insights into the pattern of these rearrangements, a near-complete genome map of a species that is less diverged from *An. gambiae* than *An. albimanus* is needed. A representative of subgenus Anopheles, *An. atroparvus*, which diverged from species of subgenus Cellia about 58 MYA, can be a suitable candidate for studying inter-arm rearrangements in malaria mosquitoes.

*Anopheles atroparvus* belongs to the Maculipennis subgroup [26], which also includes *An. artemievi, An. beklemishevi, An. daciae*, *An. labranchiae, An. maculipennis, An. martinius, An. melanoon*, *An. messeae, An. persiensis*, and *An. sacharovi.* Four species from the Maculipennis subgroup, *An. atroparvus, An. labranchiae, An. messeae,* and *An. sacharovi,* are dominant vectors of malaria in Europe and the Middle East [27]. The distribution of *An. atroparvus* ranges from Great Britain to Europe to North Caucasus in Russia but avoids some Mediterranean regions, such as Southern Italy, Greece, and Turkey. The risk of malaria transmission by *An. atroparvus* exists in Eastern Spain [28, 29], Portugal [30], South France [31], and England [32]. The epidemiological importance of *An. atroparvus* stimulated early studies of various aspects of this species’ biology, including cytogenetics [33–35]. The first cytogenetic photomap was developed for polytene chromosomes from the salivary glands [36]*.* This map followed the nomenclature of the drawn cytogenetic maps for other species from the Maculipennis group [37]. Chromosomes were numbered in the order of increasing size. The longer and shorter arms were named as the right and left arms, respectively. Based on the banding patterns, the chromosomes were divided into 39 numbered regions. The banding pattern of the *An. atroparvus* chromosomes was considered as standard, and the map was used to study chromosomal evolution and phylogenetic relationships based on overlapping chromosomal inversions among the Palearctic members of the Maculipennis group [38]. Inversion polymorphism is rarely detected in natural populations of *An. atroparvus*. For example, only one paracentric inversion on the 3L arm was found at low frequency in eastern Europe [38].

The genome of the EBRO strain of *An. atroparvus* has been sequenced as part of the 16 *Anopheles* Genomes Project [24]. The *An. atroparvus* genome assembly was made from 101-bp paired-end Illumina HiSeq2000 reads generated from three libraries: a 180-bp insert “fragment” library, a 1.5-kb “jump” library, and a 38-kb fosmid scale Illumina (“fosill”) library. The draft genome assembly of this species consisted of 1371 scaffolds with an N50 scaffold size of 9,206,694 bp and a total assembly size of 224,290,125 bp. The fragmented assembly of *An. atroparvus* did not allow studies that require chromosome-level genomic data such as the analysis of chromosomal rearrangements. In the effort to anchor genomic scaffolds to chromosomes, a new cytogenetic photomap for ovarian nurse cell chromosomes of *An. atroparvus* has been developed [21]. This map was constructed from high-resolution phase-contrast digital images of chromosomes, which were straighten to facilitate physical genome mapping. A previous mapping assigned 88.82 Mb of genomic scaffolds to polytene chromosomes, which constitute ~ 40% of the *An. atroparvus* genome assembly [21, 24].

To study the pattern of genomic rearrangements in the genus *Anopheles*, we upgraded the previously developed cytogenetic map [21] and physically mapped 89.6% of the *An. atroparvus* genome assembly to the chromosomes. Our comparative analysis of the *An. atroparvus*, *An. gambiae,* and *An. albimanus* physical genome maps led to the discovery of partial-arm reciprocal translocations. In addition, we characterized the rates of the rearrangements among the species and identified large conserved synteny blocks within chromosomal arms.

## Methods

### Mosquito colony maintenance and ovary preservation

*Anopheles atroparvus* mosquitoes were obtained from a laboratory colony hosted by Tomsk State University, Russia. The laboratory colony was maintained in the insectary at 27 °C, with a 12-h cycle of light and darkness. To obtain half-gravid females, mosquitoes were blood fed on defibrinated sheep blood using artificial bloodfeeders. Approximately 30–36 h post-blood feeding, ovaries were pulled out of abdomens and fixed in Carnoy’s solution (3: 1, ethanol: glacial acetic acid by volume). Ovaries were preserved in the fixative solution from 24 h up to 1 month at − 20 °C.

### Chromosome preparation

A single ovary from one pair was used for one preparation of ovarian nurse cell chromosomes. Ovaries were held for 5 min in a drop of 50% propionic acid, where they were macerated and squashed on a slide. The quality of preparations was checked under an AxioImager A1 microscope (Carl Zeiss, OPTEC LLC, Novosibirsk, Russia). High-quality preparations were then flash-frozen in liquid nitrogen. After removing coverslips, preparations were dehydrated in an ethanol series (50, 70, and 96%), air-dried, and stored for up to 3 months at room temperature.

### Cytogenetic map upgrade

Chromosome images were observed using a phase-contrast AxioImager A1 microscope with an attached CCD camera MRc5 using AxioVision Version 4.7.1 software (Carl Zeiss, OPTEC LLC, Novosibirsk, Russia). Images were combined, straightened, shaped, and cropped using Adobe Photoshop CS as described elsewhere [39]. Most of the chromosome images, arm naming, and borders of divisions and subdivisions were taken from the published cytogenetic map [21]. New improved images were incorporated into some subtelomeric and pericentromeric regions, and a reverse order of lettered subdivisions was used for arms 2L and 3L.

### Probe preparation and fluorescence in situ hybridization

Gene-specific primers were designed to amplify unique exon sequences from the beginning and end of each scaffold using PRIMER-BLAST software available at NCBI (http://www.ncbi.nlm.nih.gov/tools/primer-blast/). The primer design was based on gene annotations from the AatrE1 genome assembly available at VectorBase (https://www.vectorbase.org/organisms/anopheles-atroparvus/ebro/aatre1). Polymerase chain reaction (PCR) was performed in the presence of a 1× PCR buffer (SibEnzyme Ltd., Novosibirsk, Russia), 2.5 mM MgCl_2_ (SibEnzyme Ltd., Novosibirsk, Russia), 0.2 mM dNTP (Thermo Fisher Scientific, Waltham, MA, USA), and 0.02 u/μl Taq Polymerase (SibEnzyme Ltd., Novosibirsk, Russia). Amplified fragments were labeled using random primers. 25 μl of labelling reaction contained 50 ng of DNA, 1× Klenow buffer (Thermo Fisher Scientific, Waltham, MA, USA), 44 ng/μl Exo-Resistant Random Primer (Thermo Fisher Scientific, Waltham, MA, USA) 0.1 mM dATP, dGTP, dCTP and 0.015 mM dTTP, 0.016 mM TAMRA-5-dUTP or Biotin-11-dUTP (Biosan, Novosibirsk, Russia), and 5 u Klenow fragment (Thermo Fisher Scientific, Waltham, MA, USA). The required amounts of DNA, Klenow buffer, and Random Primer were mixed, brought up to 12 μl with water, and heated to 95 °C for 5 min in a thermocycler. The solution was chilled on ice, and appropriate amounts of nucleotides, Klenow fragment, and water were added to the final volume of 25 μl. The reaction mixture was incubated at 37 °C for 18 h. Fluorescence in situ hybridization (FISH) was performed using a previously described standard protocol [39]. Biotin-labelled DNA probes were detected by Avidin conjugated with FITC (Sigma-Aldrich, St. Louis, MO, USA) diluted 1:100 with blocking solution (1% Bovine Serum Albumin (Sigma-Aldrich, St. Louis, MO, USA), 1× PBS with 0.1% Tween-20) for 30 min at 37 °C. The rest of the unbound detector was washed with 1× PBS with 0,1% Tween-20 three times for 5 min each at room temperature. Overall chromosome painting was performed by DAPI in the antifade mounting medium Vectashield (Vector Laboratories, Inc., Burlingame, CA, USA).

### Synteny-based scaffolds adjacencies

Several potential scaffold adjacencies with no gaps were identified during physical mapping. For “gluing” *An. atroparvus* genomic scaffolds, several dozen genes from the candidate scaffolds’ ends were selected. Orthologous genes for these genes in the *An. gambiae* and *An. albimanus* genomes were identified using BioMart (http://biomart.vectorbase.org/biomart/martview/). If orthologs for all *An. atroparvus* genes from candidate scaffolds’ ends were located continuously in single scaffolds of *An. gambiae* and *An. albimanus*, *An. atroparvus* scaffolds were considered adjacent to each other without gaps.

### Identification of conserved synteny blocks and rearrangements

The BioMart service (http://biomart.vectorbase.org/biomart/martview/) was used to extract the set of all annotated orthologous genes for *An. atroparvus* (AatrE1 genome assembly v.86.1), *An. albimanus* (AalbS2 genome assembly v.86.2), and *An. gambiae* (AgamP4 genome assembly v.86.4). These data were prepared using R and RStudio IDE to generate an input file for GRIMM-Synteny v. 2.02 [40]. During the preparation of the data, coordinates of the *An. atroparvus* genes within genomic scaffolds were converted into coordinates on the *An. atroparvus* chromosomes. GRIMM-Synteny takes coordinates of orthologous genes of species and forms syntenic blocks based on specified parameters, such as minimum block size and gap threshold (a maximum distance between genes in a synteny block). Synteny blocks obtained by GRIMM-Synteny were visualized using genoPlotR [41]. The Multiple Genome Rearrangement (MGR) program [42] was used to determine the number and types of rearrangements that caused the synteny block reshuffling among three species. To run the random model for synteny blocks, we obtained the sample of random breaks by generating (N-1)*2 random numbers from 0 to *M*, where *N* and *M* are a mean number of synteny blocks and a mean length of genomes (base pairs) in three species, respectively. We arranged all coordinates of random breaks ascending and measured the length between each break. To simulate random distances between synteny blocks, we randomly removed one half of the samples. Eventually, we obtained a sample of lengths for *N* random synteny blocks.

## Results

### An upgraded map for ovarian nurse cell polytene chromosomes of *An. atroparvus*

The polytene chromosome complement of *An. atroparvus* consists of five arms: X, 2R, 2L, 3R, and 3L. The karyotype from female ovarian nurse cells is represented by the smallest sex chromosome X, intermediate metacentric chromosome 2, and the largest submetacentric chromosome 3 (Table 1). We observed no polymorphic inversions in the laboratory strain of *An. atroparvus.*

**Table 1:** Measurements of *An. atroparvus* ovarian nurse cells polytene chromosomes

Parameter	Chromosome X	Chromosome 2	Chromosome 3
Average length, μm	131.4	713.1	757.4
Relative length, %	8.3	44.4	47.3
Centromere index, %	0.0	46.0	38.4

**Fig. 1:**
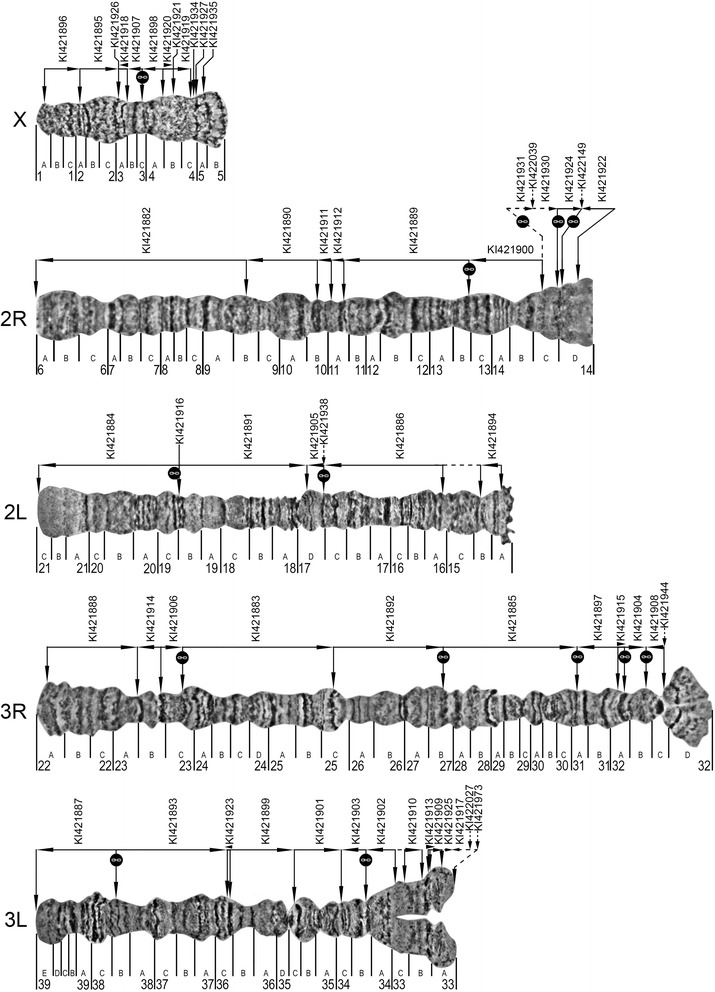
A high-resolution cytogenetic map and physical genome map for *An. atroparvus.* Numbered divisions and lettered subdivisions are shown below the chromosome images. Horizontal lines and arrows indicate the order and orientation of genomic scaffolds. The names of genomic scaffolds are shown above horizontal lines. The start and end positions of the genomic scaffolds are shown by vertical arrows corresponding to mapped FISH probes. The dashed horizontal line in 2L:15B-16A indicates a predicted adjacency of scaffolds KI421884 and KI421886. The “link” signs mark the scaffolds’ adjacencies inferred from the analysis of synteny

Here we upgraded our previously published high-resolution chromosome map for *An. atroparvus* from ovarian nurse cells [21]. We replaced subtelomeric and pericetromeric regions of arms 2L and 2R with images that have more distinct, clear banding patterns. The previous map had the order of numbered divisions and lettered subdivisions on the left arms of autosomes in the opposite order. This system was inherited from the older map [36], but it was not convenient for the genome mapping. Therefore, we reversed the order of lettered subdivisions in chromosome arms 2L and 3L (Fig. 1).

### A physical map for the *An. atroparvus* genome

**Fig. 2:**
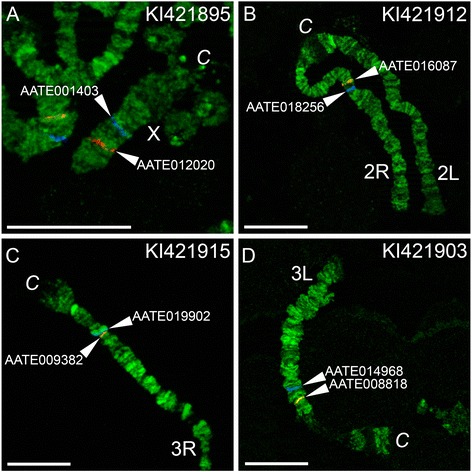
Physical mapping of genomic scaffolds to *An. atroparvus* chromosomes by FISH. The positions of the red and blue signals show the beginning and the end of the genomic scaffolds KI421895 (**a**), KI421912 (**b**), KI421915 (**c**), and KI421903 (**d**) on chromosomes X, 2R, 3R, and 3L, respectively

In this study, we physically mapped 39 genomic scaffolds to polytene chromosomes of *An. atroparvus* using FISH of gene probes (Fig. 1). Positions and adjacencies of 10 additional scaffolds were predicted based on synteny information; nine of them were also oriented. Together with previously mapped seven scaffolds [21, 24], a total of 56 scaffolds have been anchored to the *An. atroparvus* chromosomes (Additional file 1: Table S1). The mapped scaffolds constitute 200,912,972 bp or 89.6% of the total *An. atroparvus* genome assembly. To orient scaffolds using FISH, two genes, one from the beginning of the scaffold and the other from the end of the scaffold, were labeled by fluorescent dyes of different colors and hybridized to polytene chromosomes simultaneously (Fig. 2). Probes from the same genomic scaffolds were found only on the same chromosome arms, suggesting that there are no sequence misassemblies in the *An. atroparvus* genome. All probes produced clear, unique signals, and they were successfully placed onto the cytogenetic map based on the banding patterns (Fig. 1). We considered neighboring scaffolds adjacent if two FISH probes from different scaffolds hybridized to the same site on chromosomes (Additional file 2: Table S2). Our analysis of conserved synteny between genomes of *An. atroparvus* and both *An. albimanus* and *An. gambiae* also helped to juxtapose pairs of scaffolds and link them together without cytogeneic gaps. For instance, we “glued” five pairs of scaffolds on the 3R arm. Also, small gaps in regions 14C of the 2R arm, 32C of the 3R arm, and 33B of the 3L arm were filled by small scaffolds based on the synteny information (Fig. 1). The remaining unmapped 1315 scaffolds make up 23,377,153 bp or 10.4% of the total genome assembly. The unmapped portion of the genome is expected to be distributed throughout the regions of pericentromeric and intercalary heterochromatin, which have no clear banding pattern. For example, the pericentromeric heterochromatin in region 5B of the X chromosome and region 32D of the 3R arm, as well as the intercalary heterochromatin in region 15B-16A of the 2L arm, remain uncovered by the genomic scaffolds.

### Chromosome arm comparison and inter-arm rearrangements

**Fig. 3:**
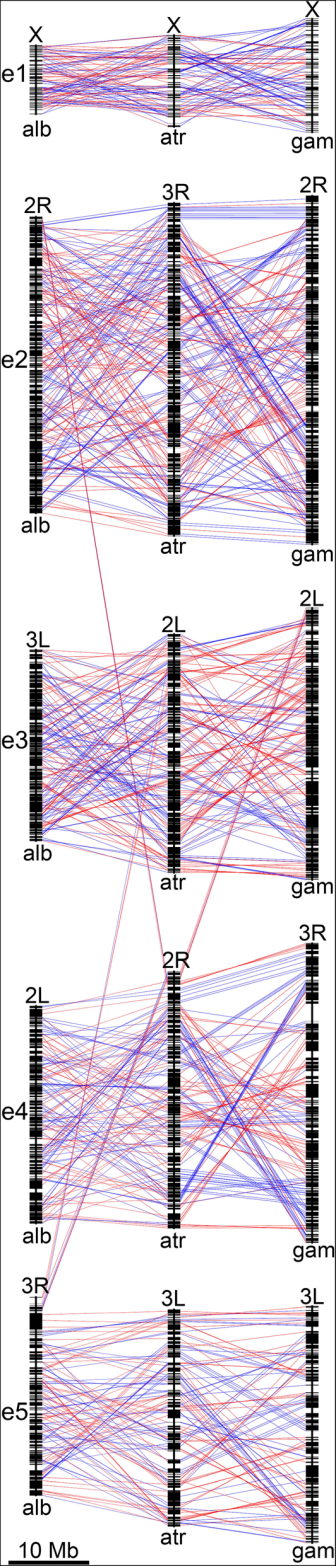
A plot of genome rearrangement among three species of *Anopheles*. Five chromosomal arms of *An. albimanus* (alb), *An. atroparvus* (atr)*,* and *An. gambiae* (gam) are shown. Chromosome elements are labeled as e1 - e5. Black rectangles along the chromosomes represent conserved synteny blocks. Blocks conserved among species are connected by blue lines if they are in the same orientation and by red lines if they are in reverse orientation relative to *An. atroparvus* blocks, which were chosen as standard. Centromeres are on the top of each element. The pericentromeric region of the *An. atroparvus* 2R arm (e4) exchanged genomic material with e2, e3, and e5, and the pericentromeric region of the *An. atroparvus* 2L (e3) exchanged genomic material with e5 of other species during evolution of *Anopheles*

To identify chromosomal rearrangements in malaria mosquitoes, the genome of *An. atroparvus* was aligned against the chromosomally-anchored genome assemblies of *An. gambiae* and *An. albimanus* [25, 39, 43]. We used GRIMM-Synteny to identify conserved synteny blocks shared among the three species. Our parameters for the algorithm required at least two orthologous genes within a synteny block (in order to exclude single-gene transpositions) and the gap size, which is a maximum distance between genes within a synteny block, equal to 115 kb. We identified conserved synteny blocks of average length equal to 143 kb, which is in agreement with the previous study [24]. We consider chromosomal arms as elements that are homologous across all species [11, 12]. We found that the majority of the orthologous genes are located on the same chromosome element across species, although in a different order. Likewise, the majority of rearrangements takes place within the chromosomal arm, but several exceptions are apparent. The pericentromeric region of e4 in *An. atroparvus* (arm 2R) contains several synteny blocks that correspond to e2 (2R), e3 (3L), e5 (3R) of *An. albimanus,* and e3 (2L) of *An. gambiae.* Also, the pericentromeric region of e3 in *An. atroparvus* (arm 2L) contains synteny blocks that correspond to e5 (3R) of *An. albimanus* (Fig. 3).

**Fig. 4:**
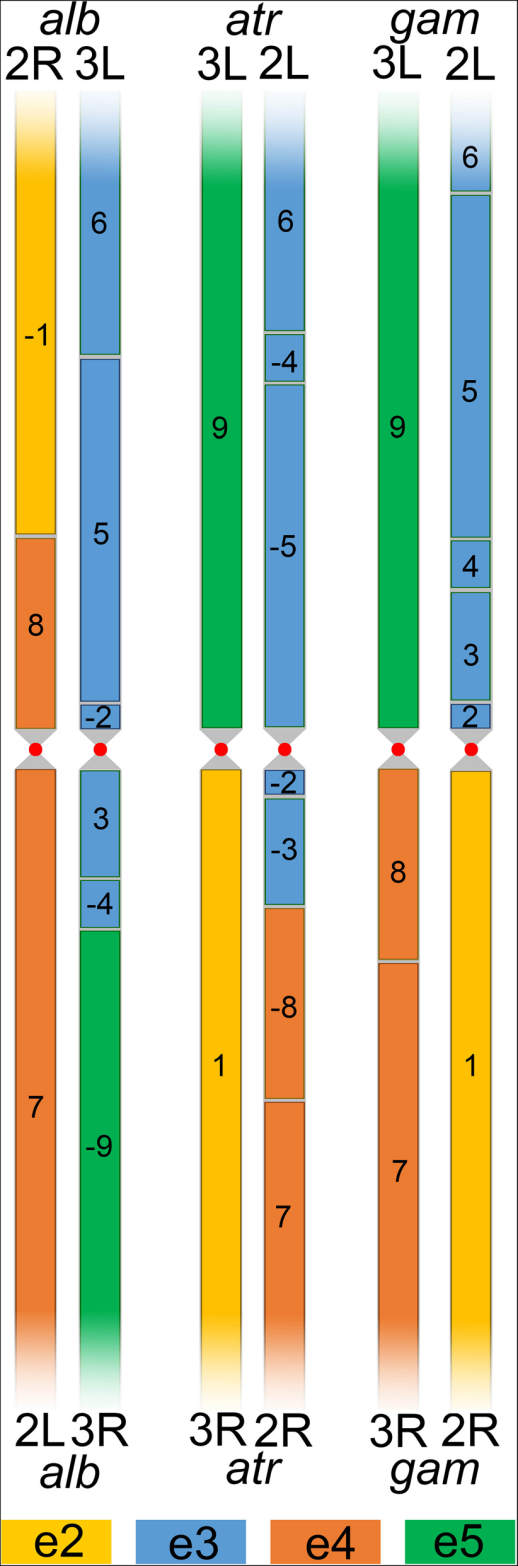
A scheme of distribution of nine major conserved synteny blocks in the pericentromeric regions of the autosomes of *An. atroparvus (atr)*, *An. albimanus (alb)*, and *An. gambiae (gam).* Centromeres are represented by red circles. The lengths of synteny blocks are not in scale

To investigate the syntenic relationships between chromosome elements of different species, we identified synteny blocks in the pericentromeric regions by BLAST. If paracentric inversions are ignored, nine major synteny blocks can be identified among *An. atroparvus*, *An. gambiae,* and *An. albimanus.* Five of these blocks changed their chromosome element position during evolution (Additional file 3: Table S3). The centromere position with respect to some of these blocks changes among species indicating inter-arm rearrangements (Fig. 4). We calculated the numbers and determined types of rearrangements between chromosome elements using the MGR program [42], which uses the same algorithm as GRIMM. The following orders and orientations of conserved blocks were used as an input for the MGR run, where the symbol $ indicates the end of each chromosome element.

- >*An. gambiae*
- 1 $ 2 3 4 5 6 $ 7 8 $ 9 $
- >*An. atroparvus*
− 1 $ -9 $ 6–4 -5 $ -2 -3 -8 7 $
- >*An. albimanus*
− 1 8 $ 7 $ 6 5–2 $ 3–4 -9 $

The MGR program reconstructs a putative ancestral karyotype, determines the types of rearrangements, creates a phylogenetic tree, and implements an algorithm that minimizes the sum of the rearrangements over all edges of the phylogenetic tree (Additional file 4: Figure S1A). Accordingly, the *An. gambiae* elements have a close resemblance with the putative ancestral karyotype, as they differ by only three paracentric inversions and have a whole-arm synteny preservation (Additional file 4: Figure S1B). In contrast, the *An. atroparvus* chromosomes differ from the ancestral karyotype by two partial-arm translocations and one paracentric inversion (Additional file 4: Figure S1C), while the *An. albimanus* accumulated three partial-arm translocations and one paracentric inversion after the divergence from the ancestral species (Additional file 4: Figure S1D). In pair-wise comparisons, *An. gambiae* and *An. atroparvus* differ by two partial-arm translocations and three paracentric inversions (Additional file 4: Figure S1E), *An. gambiae* and *An. albimanus* differ by three partial-arm translocations and three paracentric inversions (Additional file 4: Figure S1F), while *An. atroparvus* and *An. albimanus* differ by five partial-arm translocations and one paracentric inversion (Additional file 4: Figure S1G).

The lengths of the conserved synteny blocks involved in partial-arm translocations and immediately adjacent to the centromeres vary from 5031 bp (block 2 in *An. albimanus*) to 2,546,887 bp (block 5 in *An. atroparvus*) (Additional file 3: Table S3). This means that breakpoints of partial-arm translocations occurred in close proximity to the centromere. However, genes of some blocks, which changed their chromosome element position during evolution, can also be found far away from the centromere: 9.8 Mb in *An. gambiae* and 13.8 Mb in *An. albimanus.* This observation suggests that paracentric inversions moved the genes along chromosomal arms after the partial-arm translocations occurred.

### Conserved synteny blocks in genus *Anopheles*

We calculated the number of conserved synteny blocks for every chromosome element of *An. albimanus*, *An. atroparvus,* and *An. gambiae* in pairwise comparisons (Table 2). The number of synteny blocks corresponds with phylogenetic relations between the three species [24]. Accordingly, the smallest number of synteny blocks was observed between *An. atroparvus* and *An. gambiae*, which have the shortest divergence time of about 58 MYA. In agreement with previous studies [15, 23, 24], we observed enrichment of the number of conserved synteny blocks in the X chromosome (e1) (Table 2), which comprises only 8.3% of the total chromosome length, compared with autosomes (Table 1).

**Table 2:** Numbers of conserved synteny blocks in pairwise comparisons of *An. albimanus*, *An. gambiae,* and *An. atroparvus*

Species compared	e1	e2	e3	e4	e5	Total number of blocks
*An. albimanus* / *An. gambiae*	137	159	114	97	79	586
*An. atroparvus* / *An. albimanus*	105	132	66	85	52	440
*An. atroparvus* / *An. gambiae*	94	78	43	37	34	286

**Fig. 5:**
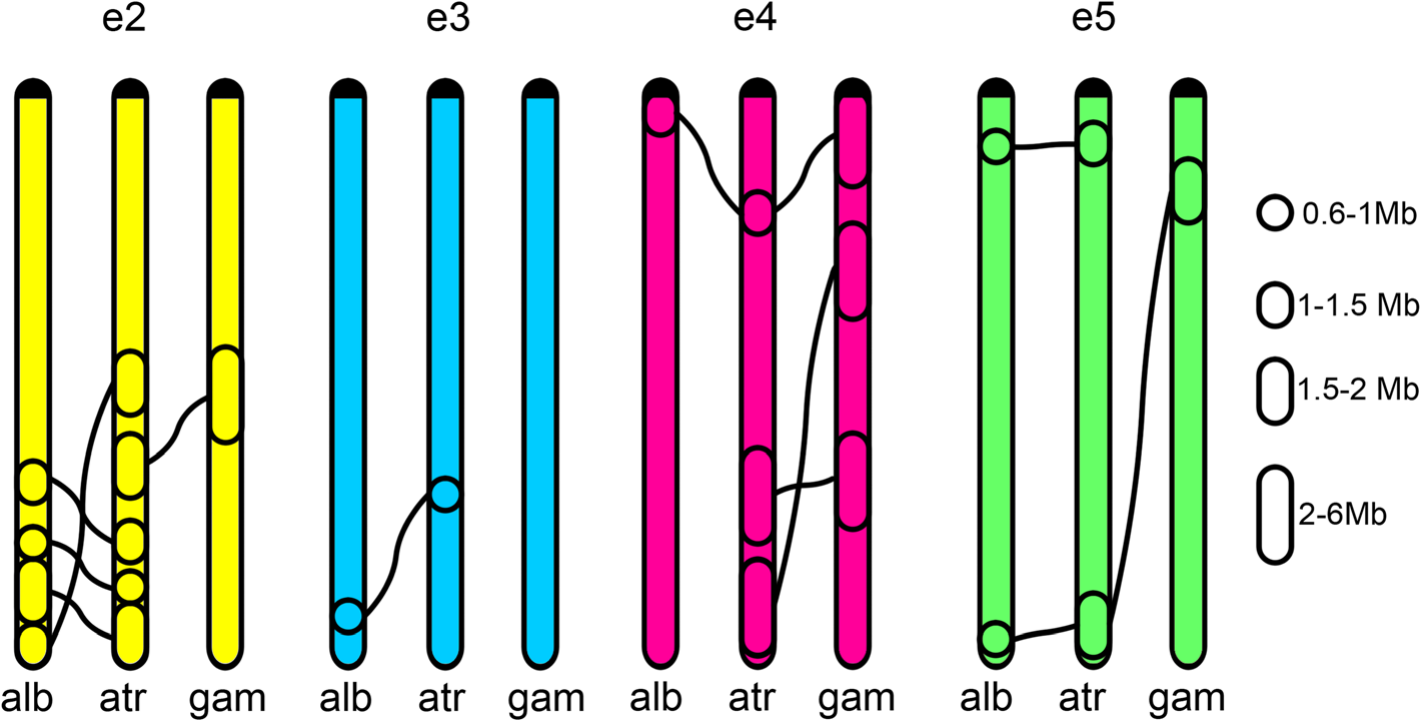
Distribution of the largest conserved synteny blocks in pair-wise comparison within the autosomal elements e2-e5. The dark caps on the top of the arms represent centromeres alb – *An. albimanus*, atr – *An. atroparvus*, and gam – *An. gambiae*

**Fig. 6:**
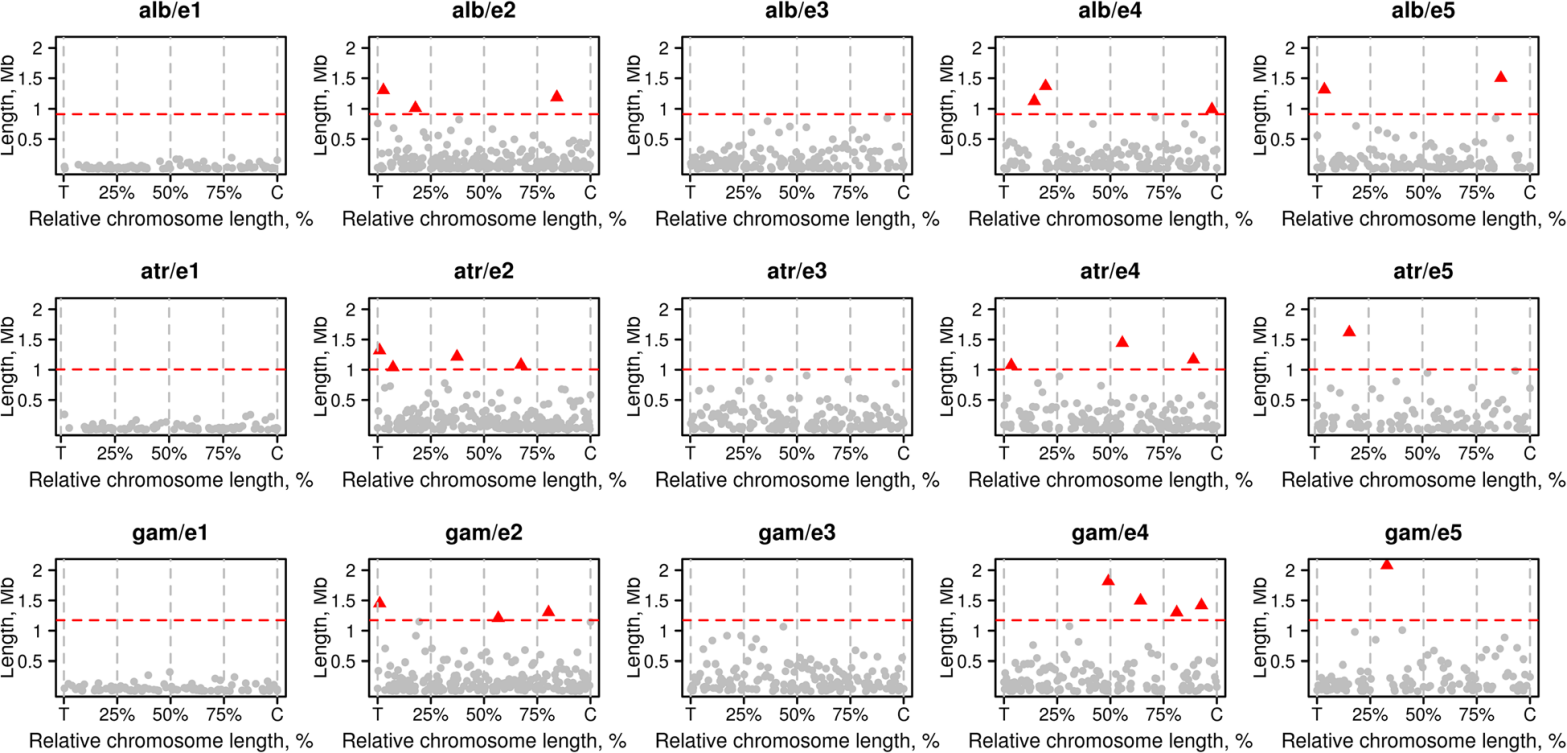
Chromosomal distribution of the largest conserved synteny blocks in three-species comparisons. Red triangles represent the length of synteny block above 99 percentile. alb – *An. albimanus*, atr – *An. atroparvus*, gam – *An. gambiae,* T – telomere, and C – centromere

We identified and localized the largest conserved synteny blocks in pairwise comparisons of autosomes, *An. albimanus*: *An. atroparvus* and *An. atroparvus*: *An. gambiae,* each time excluding the third species from the comparison (Fig. 5). This analysis demonstrated how large conserved blocks are distributed along the chromosome length. It was clear that in most cases the largest synteny blocks avoided being in the middle of the chromosomal arms, except e2 of *An. atroparvus* and *An. gambiae*. A similar trend was observed in a three-species comparison, *An. albimanus*: *An. atroparvus*: *An. gambiae*. Out of the 24 largest blocks, 17 are located either in the first (telomeric) or in the last (centromeric) quadrants of the chromosome arms. Only 7 of the largest blocks are located in the two middle quadrants of the chromosome arms (Fig. 6). As expected, lengths of the largest conserved synteny blocks are smaller than that in pair-wise comparisons (Table 3).

**Table 3:** Lengths of the largest conserved synteny blocks in a three-species comparison among *An. atroparvus*, *An. gambiae*, and *An. albimanus*

Species	Minimum length of blocks from the 99 percentile	Mean length of the largest synteny blocks	Maximum length of the largest synteny blocks
*An. albimanus*	911,714	1,226,232	1,504,168
*An. atroparvus*	1,005,254	1,245,690	1,618,976
*An. gambiae*	1,173,026	1,509,795	2,078,641

**Fig. 7:**
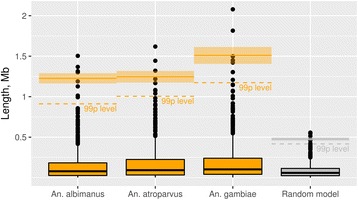
Observed lengths of synteny blocks in *An. albimanus*, *An. atroparvus*, and *An. gambiae* and calculated lengths of synteny blocks under the random breakage model. Dashed orange and grey lines indicate 99 percentiles in each sample. Solid-orange and -grey rectangles below the 99-percentile level denote the mean lengths of the synteny blocks. Translucent- orange and -grey rectangles above the 99-percentile level denote the standard error of the mean for the lengths of the large synteny blocks

Our simulation shows that these blocks cannot exist under a random breakage model (Fig. 7). Therefore, they are likely the result of functional or structural constraints to breakage.

### Rates of inversion fixation in malaria mosquitoes

Using the GRIMM-Synteny program [44], we calculated the number of fixed inversions in each chromosome element between *An. atroparvus*, *An. gambiae*, *An. albimanus,* and the putative ancestral *Anopheles* species (Table 4). As in the case with the number of conserved synteny blocks (Table 2), we observed enrichment of rearrangements in the X chromosome (e1) compared with autosomes.

**Table 4:** Fixed inversions between *An. atroparvus*, *An. gambiae*, *An. albimanus*, and the putative ancestral *Anopheles* species

Species	e1	e2	e3	e4	e5	Total
*An. gambiae*	65	62	47	30	33	139
*An. atroparvus*	32	27	15	19	10	61
*An. albimanus*	70	104	75	77	49	256

We also calculated the minimum number of paracentric inversions in pair-wise comparisons of *An. atroparvus*, *An. gambiae,* and *An. albimanus.* The number of inversions between *An. atroparvus* and *An. gambiae* was less than the number of inversions between *An. atroparvus* and *An. albimanus,* which is in concordance with their phylogenetic relations [24]. To calculate the number of rearrangements/Mb/MY for these species, we used the average length of the mapped portion of the genome assembly to each chromosomal arm of a particular pair of species and the divergence time between them. In all species pairs, the gene order on the X chromosome (e1) evolved faster than that on the autosomes (Table 5).

**Table 5:** Rearrangement per Mb per MY between pairs of species for each chromosome element

Species pairs	Number of rearrangements	Average length of the genome assembly	Divergence, MY	e1	e2	e3	e4	e5	Genome average
*An. atroparvus / An. gambiae*	309	248,699,585	58	0.077	0.025	0.026	0.019	0.020	0.043
*An. atroparvus / An. albimanus*	465	198,814,682	100	0.056	0.022	0.022	0.021	0.016	0.047
*An. albimanus / An. gambiae*	568	223,224,142	100	0.076	0.033	0.034	0.029	0.025	0.051

## Discussion

### Comparison of the *An. atroparvus* genome map with mapped assemblies of other anophelines

In this study, we developed a detailed physical map of the *An. atroparvus* genome with 89.6% of the sequenced assembly anchored to chromosomes (Fig. 1, Additional file 1: Table S1). After *An. albimanus,* the *An. atroparvus* genome has the second genome mapping coverage among Anophelinae genome assemblies (Table 6). The recently developed AalbS2 genome assembly has 98.2% of the total genome mapped to the *An. albimanus* chromosomes

**Table 6:** Comparison of genome assemblies and mapped genomes for malaria mosquitoes

Species	Original assembly	Total scaffolds	Mapped scaffolds	Scaffold N50, bp	Total length, Mb	Mapped length, Mb	Mapped, %	Reference
*An. albimanus*	AalbS1	204	40	18,068,499	170.5	167.4	98.2	[25]
*An. atroparvus*	AatrE1	1371	56	9,206,694	224.3	200.9	89.6	This study
*An. arabiensis*	AaraD1	1214	50	5,604,218	246.6	216.3	87.7	[56]
*An. gambiae*	AgamP3	8	5	49,364,325	273.1	230.2	84.3	[43]
*An. stephensi*	AsteI2	23,371	86	1,591,355	221.3	137.14	62	[14]
*An. stephensi*	AsteS1	1100	101	837,295	225	92.83	41	[24]
*An. sinensis*	AsinC2	9592	52	814,231	220.8	79.3	35.9	[23]
*An. funestus*	AfunF1	1392	103	671,960	225.2	79.0	35.1	[24]

(https://www.vectorbase.org/organisms/anopheles-albimanus/stecla) [25]. For comparison, the physically mapped portion of the *An. gambiae* AgamP4 assembly is equal to 84.3% of the total sequenced genome [39, 43]. However, *An. gambiae* has the highest density of chromosomally mapped DNA markers among mosquitoes. The *An. stephensi* AsteI2 [14], *An. sinensis* AsinC2 [23], and *An. funestus* AfunF1 [24] assemblies have 62, 35.9, and 35.1% of their genomes mapped chromosomes, respectively. A relatively high coverage of the physical map for *An. atroparvus* was achieved due to the good quality of the genome assembly. The AatrE1 genome assembly consists of 1371 scaffolds with an N50 scaffold size of 9.2 Mb (https://www.vectorbase.org/organisms/anopheles-atroparvus/ebro/aatre1). This N50 scaffold size is still twice less than the N50 size of 18.1 Mb for *An. albimanus* but much higher than the N50 sizes of 1.6 Mb for *An. stephensi,* 814 kb for *An. sinensis*, or 672 kb for *An. funestus.* Unlike in *An. albimanus*, our physical mapping did not detect any scaffold misassemblies in *An. atroparvus*.

### The pattern of chromosome rearrangements in malaria mosquitoes

We used the physical map developed for *An. atroparvus* in this study to analyze the chromosome rearrangements in the genus *Anopheles*. The analysis has shown that some pericentromeric regions of chromosome arms 2R (e4) of *An. atroparvus* are homologous to portions of arms 2R (e2), 3L (e3), and 3R (e5) of *An. albimanus,* and to 2L (e3) of *An. gambiae* (Figs. 3 and 4). This observation violates the previously accepted notion of the whole-arm synteny preservation in the evolution of malaria mosquitoes [11, 12, 14–24]. Our recent study identified the first case of genomic exchanges between chromosome elements that happened during the split of the *An. albimanus* and *An. gambiae* lineages [25]. Here, the detailed analysis of rearrangements among *An. atroparvus*, *An. albimanus,* and *An. gambiae* using the MGR program demonstrated that autosomal elements exchange their genetic material via multiple partial-arm translocations. However, cases of arm integrity preservation were also found in the evolution of malaria mosquitoes. For example, elements 2 and 5 of *An. gambiae* and *An. atroparvus* are fully syntenic, and they changed their associations within chromosomes via whole-arm translocations (Fig. 4). Moreover, all chromosome elements of *An. gambiae* are fully syntenic with chromosome elements of the putative ancestral *Anopheles* karyotype. Our previous comparative genomic analysis between *An. gambiae* and *An. stephensi* indicated that the whole-arm synteny is preserved between these two representatives of subgenus Cellia [14]. Our present study suggests that the integrity of ancestral chromosome elements was broken in subgenera Anopheles and Nyssorhynchus, but not in Cellia. This observation may point to the existence of structural or functional lineage-specific constraints to karyotype evolution in subgenus Cellia.

This study demonstrates distinct features of karyotype evolution in anopheline mosquitoes if compared with fruit flies. The diploid number of chromosomes can change in the evolution of the genus *Drosophila*, as they can undergo centric fission or fusion. For example, Muller element F has fused to Muller E in *D. willistoni* [45, 46]. Also, Muller element D fused with Muller A to become X-linked in *D. pseudoobscura* [46, 47]. The integrity of Muller elements can be broken by pericentric inversions. A pericentric inversion has shuffled genes between Muller elements A and D in *D. pseudoobscura* and *D. persimilis* [8, 47]. A shared pericentric inversion has occurred between Muller B and C in *D. erecta* and *D. yakuba* [48, 49]. In contrast, the diploid number of chromosomes is always three in *Anopheles*, but autosomal arm associations changed multiple times during mosquito evolution. The integrity of chromosome elements can be broken by partial-arm translocations. However, unlike *Drosophila*, the sex chromosome (e1) of malaria mosquitoes does not exchange or merge its genetic material with autosomes via translocations. However, gene retrotransposition rates from the X chromosome to autosomes even exceed those in fruit flies [24]. Still, the common feature of chromosome evolution shared between mosquitoes and flies is the abundance of paracentric inversions.

### The rate of chromosome rearrangements in malaria mosquitoes

Our GRIMM analysis of paracentric inversions reaffirms the phylogenetic relationships among mosquito lineages. The *An. albimanus* lineage is one of the earliest radiations within genus *Anopheles*, which happened about 100 MYA, while *An. atroparvus* and *An. gambiae* were part of the same lineage until 58 MYA [24, 50]. We found that the number of conserved synteny blocks between *An. atroparvus* and *An. gambiae* was 1.5–2 times smaller than the number of conserved synteny blocks between either *An. atroparvus* or *An. gambiae* and *An. albimanus* (Table 2)*.* The obtained values of rearrangement rates between *An. atroparvus* and *An. gambiae* or *An. albimanus* and *An. gambiae* were generally lower than those estimated in a previous study [24]. This difference can be explained by the larger portion of the mapped genome in *An. albimanus* [25] and *An. atroparvus* used in this study. Our study confirmed previous observations [15, 23, 24] that the X chromosome has higher rates of rearrangement than do autosomes (Table 5). The phenomenon of the rapid fixation of X chromosome rearrangements could indicate that inversions on the X chromosome are underdominant when in heterozygote, as was theoretically predicted earlier [51].

Despite intensive reshuffling of the chromosomal segments during the evolution of malaria mosquitoes (Fig. 3), certain forces maintain long linkages of genes. Our simulation suggests that large conserved synteny blocks could not be preserved in evolution if breakpoints are randomly distributed along the chromosomes (Fig. 7). Instead, functional or structural constraints to breakage are likely responsible for the preservation of these blocks. The adjacent groups of genes could be resistant to breakage due to co-regulation of gene expression, co-adaptation, and/or spatial position in the nucleus that restrict chromosome rearrangements. However, the experimental disruption of one of the largest conserved genomic regions in *D. melanogaster* caused an unexpectedly small phenotypic effect, namely alteration of odorant perception by flies [52]. Moreover, this change in odorant perception did not correlate with changes in gene expression within the disrupted conserved synteny block. We observed a tendency of the largest synteny blocks to concentrate close to the ends of the chromosome arms and to avoid the middle part of the arms (Figs. 5 and 6). Subtelomeric and pericentromeric chromosome regions are located at the nuclear periphery, and perhaps this feature restricts their ability to participate in long-range rearrangements. Indeed, a study in *Drosophila* has found that the largest conserved chromosomal regions are enriched in proteins Lamin and SUUR located at the nuclear periphery [53]. According to the systematic study of 53 chromatin proteins in *Drosophila* cells, Lamin and SUUR characterize transcriptionally inert “black” type of five principal chromatin types [54]. A previous three-species comparison of physical maps within subgenus Cellia [55] revealed that the autosomal arms differ in their tolerance to the distribution of conserved gene orders. If a block on e2 was conserved between two mosquito species, it was likely disrupted in the third species. In contrast, all identified synteny blocks remained preserved on e4, suggesting the existence of arm-specific constraints to breakage [55]. However, our comparative genomic study demonstrated the highest concentration of largest conserved synteny blocks in both e2 and e4. Also, the minimum number of largest conserved synteny blocks was found not on e2 but on e3 (Figs. 5 and 6), suggesting that the dynamics of autosomal breakage differ between Cellia and other subgenera.

## Conclusions

The physical genome map developed for the European malaria vector *An. atroparvus* in this study demonstrates the power of chromosome-based assemblies for understanding genome evolution. The chromosome rearrangements identified here would be impossible to observe with routine cytogenetic techniques or with unmapped genome assemblies. We found that chromosomal arms in malaria mosquitoes can exchange their genetic material by partial-arm translocations. This observation challenges the presumed syntenic relationships of chromosomal arms among *Anopheles* species. Instead, the genetic content of a chromosome element cannot be assumed to be conserved across anophelines. Our findings suggests that the ancestral chromosome elements retain their integrity in subgenus Cellia, but not in subgenera Anopheles and Nyssorhynchus. The study supports the previously identified phenomenon of the rapid fixation of X chromosomal rearrangements. Finally, our data suggest the existence of mechanisms that govern the chromosomal arm and lineage specificity in rates of gene order disruption during the malaria mosquito evolution.

## Additional files

Additional file 1: **Table S1.** Sizes, coordinates, and chromosomal positions of mapped *An. atroparvus* scaffolds. (XLSX 52 kb)

Additional file 2: **Table S2.** Scaffold-paired adjacencies in the *An. atroparvus* genome assembly. (XLSX 14 kb)

Additional file 3: **Table S3**. Lengths of pericentromeric-conserved synteny blocks in *Anopheles*. (DOCX 18 kb)

Additional file 4: **Figure S1.** A phylogenetic tree and types of rearrangements determined by the MGR program. **A**) Reconstructed phylogenetic tree determines a minimum number of rearrangements between each modern karyotype and the putative ancestral karyotype. **B**) Number and types of rearrangements between *An. gambiae* and putative ancestral species. **C**) Number and types of rearrangements between *An. atroparvus* and putative ancestral species. **D**) Number and types of rearrangements between *An. albimanus* and putative ancestral species. **E**) Number and types of rearrangements between *An. gambiae* and *An. atroparvus.* **F**) Number and types of rearrangements between *An. gambiae* and *An. albimanus.* **G**) Number and types of rearrangements between *An. atroparvus* and *An. albimanus*. (TIF 1384 kb)

## Abbreviations

Bp: Base pair
E: Element
GRIMM: Genome Rearrangements in Man and Mouse
kb: Kilobase
Mb: Megabase
MYA: Million years ago
