# Supplementary material for: Partial-arm translocations in evolution of malaria mosquitoes revealed by high-coverage physical mapping of the Anopheles atroparvus genome
Source: BMC Genomics. 2018 Apr 23;19:278. doi: 10.1186/s12864-018-4663-4 (PMC5914054; doi:10.1186/s12864-018-4663-4)
Supplement: Supplementary file 3 — Table S3. Lengths of pericentromeric-conserved synteny blocks in Anopheles. (DOCX 18 kb) [file 12864_2018_4663_MOESM3_ESM.docx]

| Synteny block ID | Number of genes |  | *An. albimanus* | *An. atroparvus* | *An. gambiae* | Block average length, bp |
| --- | --- | --- | --- | --- | --- | --- |
| 2 | 1 | Marginal genes ID | AALB009750 | AATE000869 | AGAP004692 |  |
|  |  | Synteny block length, bp | **5,031** | **14,201** | **30,369** | 16,534 |
| 3 | 15 | Marginal genes ID | AALB009746-AALB007503 | AATE010427-AATE011237 | AGAP004775-AGAP004703 |  |
|  |  | Synteny block length, bp | **2,160,999** | 1,217,848 | 1,147,354 | 1,508,734 |
| 4 | 7 | Marginal genes ID | AALB007511-AALB007524 | AATE013921-AATE016229 | AGAP004787-AGAP004805 |  |
|  |  | Synteny block length, bp | 166,799 | 300,835 | 226,020 | 231,218 |
| 5 | 26 | Marginal genes ID | AALB004864-AALB003225 | AATE012085-AATE009040 | AGAP004827-AGAP005305 |  |
|  |  | Synteny block length, bp | 13,789,960 | **2,546,887** | 9,785,426 | 8,707,424 |
| 8 | 22 | Marginal genes ID | AALB014148-AALB002927 | AATE001155-AATE007043 | AGAP010253-AGAP010295 |  |
|  |  | Synteny block length, bp | **786,803** | 511,447 | **889,790** | 729,347 |

**Table S3**. Lengths of pericentromeric conserved synteny blocks in *Anopheles*.

Lengths of blocks adjacent to centromeres are in **bold**.
