# Supplementary figures and images for: Partial-arm translocations in evolution of malaria mosquitoes revealed by high-coverage physical mapping of the Anopheles atroparvus genome
Source: BMC Genomics. 2018 Apr 23;19:278. doi: 10.1186/s12864-018-4663-4 (PMC5914054; doi:10.1186/s12864-018-4663-4)

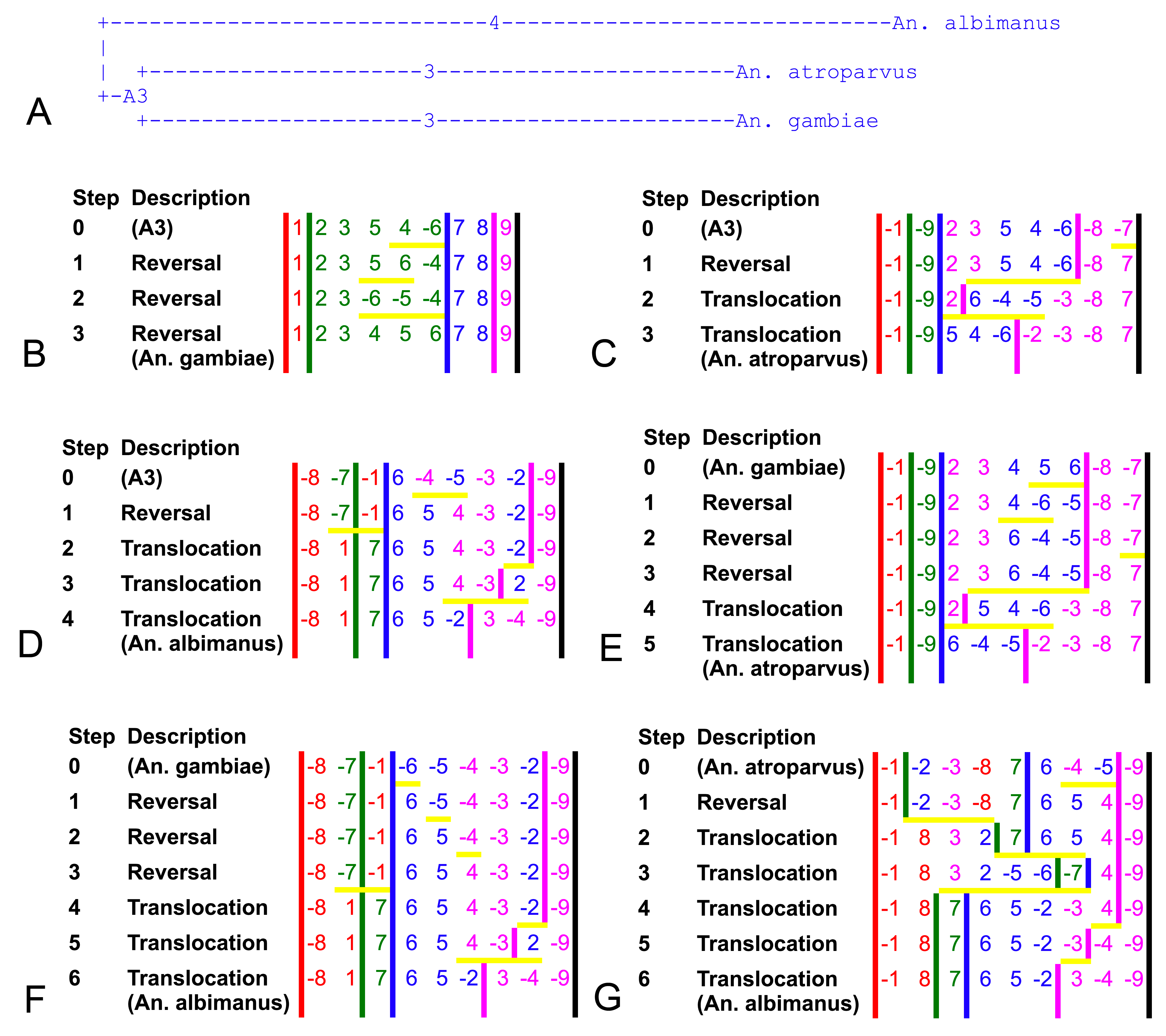

Supplement: Supplementary file 4 — Figure S1. A phylogenetic tree and types of rearrangements determined by the MGR program. A) Reconstructed phylogenetic tree determines a minimum number of rearrangements between each modern karyotype and the putative ancestral karyotype. B) Number and types of rearrangements between An. gambiae and putative ancestral species. C) Number and types of rearrangements between An. atroparvus and putative ancestral species. D) Number and types of rearrangements between An. albimanus and putative ancestral species. E) Number and types of rearrangements between An. gambiae and An. atroparvus. F) Number and types of rearrangements between An. gambiae and An. albimanus. G) Number and types of rearrangements between An. atroparvus and An. albimanus. (TIF 1384 kb) [file 12864_2018_4663_MOESM4_ESM.tif]
